# Potential in vitro anti-periodontopathogenic, anti-*Chikungunya* activities and in vivo toxicity of Brazilian red propolis

**DOI:** 10.1038/s41598-022-24776-4

**Published:** 2022-12-07

**Authors:** Nagela Bernadelli Sousa Silva, Jonathan Henrique de Souza, Mariana Brentini Santiago, Jhennyfer Rodrigues da Silva Aguiar, Daniel Oliveira Silva Martins, Rafael Alves da Silva, Igor de Andrade Santos, Jennyfer A. Aldana-Mejía, Ana Carolina Gomes Jardim, Reginaldo dos Santos Pedroso, Sergio Ricardo Ambrósio, Rodrigo Cássio Sola Veneziani, Jairo Kenupp Bastos, Regina Helena Pires, Carlos Henrique Gomes Martins

**Affiliations:** 1 Institute of Biomedical Sciences (ICBIM), Federal University of Uberlândia, Uberlândia, Brazil; 2 Institute of Biosciences, Letters and Exact Sciences (IBILCE), Sao Paulo State University, São José Do Rio Preto, Brazil; 3 Faculty of Medicine (FAMED), Federal University of Uberlândia, Uberlândia, Brazil; 4 Faculty of Pharmaceutical Sciences of Ribeirão Preto, University of São Paulo (USP), Ribeirão Preto, Brazil; 5 Technical School of Health (ESTES), Federal University of Uberlândia, Uberlândia, Brazil; 6 Exact and Technological Sciences Nucleus, University of Franca (UNIFRAN), Franca, Brazil; 7 Postgraduate Program in Health Promotion, University of Franca (UNIFRAN), Franca, Brazil

**Keywords:** Antimicrobials, Applied microbiology, Biofilms, Microbiology, Virology, Antivirals

## Abstract

Bacterial and viral infections are serious public health issue. Therefore, this study aimed to evaluate the antibacterial, antibiofilm and antiviral potential of the Brazilian Red Propolis (BRP) crude hydroalcoholic extract, fractions, and isolated compounds, as well as their in vivo toxicity. The antibacterial activity was evaluated by determining the Minimum Inhibitory Concentration and the antibiofilm activity by determining the Minimum Inhibitory Concentration of Biofilm (MICB_50_). The viable bacteria count (Log_10_ UFC/mL) was also obtained. The antiviral assays were performed by infecting BHK-21 cells with *Chikungunya* (CHIKV) *nanoluc*. The toxicity of the BRP was evaluated in the *Caenorhabditis elegans* animal model. The MIC values for the crude hydroalcoholic extract sample ranged from 3.12 to 100 μg/mL, while fractions and isolated compounds the MIC values ranged from 1.56 to 400 μg/mL.The BRP crude hydroalcoholic extract, oblongifolin B, and gutiferone E presented MICB_50_ values ranging from 1.56 to 100 μg/mL against monospecies and multispecies biofilms. Neovestitol and vestitol inhibited CHIKV infection by 93.5 and 96.7%, respectively. The tests to evaluate toxicity in *C. elegans* demonstrated that the BRP was not toxic below the concentrations 750 μg/mL. The results constitute an alternative approach for treating various infectious diseases.

## Introduction

Despite important advances in the health area, infectious diseases have constituted a serious public health issue over time^1^. One example is periodontitis, an inflammatory disease that affects tooth‐supporting apparatus and which is caused by microorganisms present in dysbiosis plaque biofilms^2^. According to Mehrotra and Singh^3^, about 2.6% of African Americans, 5% of Africans, 0.2% of Asians, 1% of North Americans, and 0.3% of South Americans have been diagnosed with periodontitis in its most severe form. Periodontal treatment is essential not only for dental parameters, but also to avoid other pathological conditions such as adverse reactions in pregnancy, cardiovascular and respiratory diseases, cancer, lupus, rheumatoid arthritis, diabetes mellitus, and chronic kidney disease^4^. Even if the illness can be treated with antibiotics, the infection can be aggravated in patients lacking treatment or in the presence of resistant periodontopathogenic bacteria^5^.

Viral diseases also burden the global health system due to lack of vaccines and approved antivirals to combat important human viruses, including the *Chikungunya Fever*, caused by the *Chikungunya* virus (CHIKV)^6^. CHIKV was identified in 2014 and has become hyperendemic in Brazil^7^, This virus causes dengue-like symptoms such as fever, fatigue, arthralgia, and polyarthralgia^8^. By April 2022, 28,291 suspected cases of *Chikungunya Fever* had been registered and five deaths had been confirmed in Brazil; another eight deaths are under investigation^9^.

According to the World Health Organization, a considerable part of the worldwide population still depends on traditional medicine and employs natural products to treat several diseases^10^. Developing countries mainly use such products. In this scenario, Brazil is a valuable source of natural products given that it possesses diverse fauna and flora^11^. Brazilian Red Propolis (BRP), a resinous material produced by *Apis mellifera* bees through the collection of the exudates of two plant species: *Dalbergia ecastaphyllum*^12,13^ and *Symphonia globulifera*^14^ has excellent potential for developing new medicines. BRP is currently one of the most produced and commercialized types of Brazilian propolis. It is mainly found in the Brazilian mangroves of the Northeast, especially in Alagoas and Bahia states^15^.

BRP is composed of 50% resin, 30% wax, 10% essential oils, 5% pollen, and 5% other compounds, including secondary metabolites like flavonoids, isoflavonoids, cinnamic acid derivatives, esters, polyprenylated benzophenones, and some terpenes, which are considered the main biologically active constituents of this type of propolis^16^. The molecules isolated from BRP do not occur in any other type of propolis, which makes them rare and unique natural products^17^. Variations of this composition have been observed between locations. Some studies revealed that compounds such as formononetin and isoliquiritigenin are the most abundant in samples of Alagoas^18^. Instead, in “Canavieiras” sample, vestitol, neovestitol, medicarpin, and polyprenylated benzophenones have been identified as the main compounds and^17^. In this sense, BRP has been reported to possess antibacterial^15,18–20^ antiparasitic^21–27^, and antiviral activities^28^.

Considering the lack of treatment options for periodontitis and CHIKV infection, we have hypothesized that BRP and its isolated compounds are a promising candidate for treating these diseases. To the best of our knowledge, there are no data on the BRP antiviral action against CHIKV, and few studies have reported on its antibacterial action against periodontopathogenic bacteria^13,18,28–30^. The use of BRP as a therapeutic option could reduce the use of antibiotics in periodontitis cases and become a novel antiviral strategy against CHIKV^28^.

This study aimed to evaluate the in vitro antibacterial, antibiofilm, and antiviral potential of the BRP crude hydroalcoholic extract, fractions, and isolated compounds, as well as their toxicity in an in *vivo* model.

## Results

### BRP crude extract characterization

**Figure 1:**
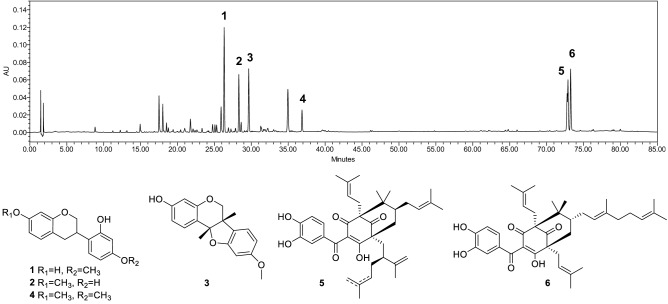
Chromatographic profile of Brazilian red propolis extract and chemical structures of their main compounds. Numbers correspond to: vestitol (1); neovestitol (2); medicarpin (3); 7-*O*-methylvestitol (4); guttiferone E/xanthochymol ^59^; and oblongifolin B (6).

The chromatographic analysis revealed the presence of isoflavanes (vestitol, neovestitol, 7-*O*-methylvestitol), pterocaparns (medicarpin), and polyprenylated acylphloroglucinols (a mixture of guttiferone E/xanthochymol, and oblongifolin B) (Fig. 1), as main compounds of the BRP. The chromatographic profile of the fractions revealed the prominent presence of polyprenylated acylphloroglucinols on the hexane fraction, whereas the dichloromethane, ethyl acetate, and *n*-butanol fractions were composed mainly of isoflavanes (see Supplementary Figure S1).

### Minimum inhibitory concentration of the BRP crude hydroalcoholic extract, fractions, and isolated compounds

Tables 1 and 2 show the MIC results for the crude hydroalcoholic extract, fractions and isolated compounds against periodontal bacteria included in the study. The MIC values for the crude hydroalcoholic extract sample ranged from 3.12 to 100 μg/mL, for the dichloromethane fraction from 1.56 to 200 μg/mL, ethyl acetate from 12.5 to 400 μg/mL, hexane from 3.12 to 400 μg/mL, and *n*-Butanol from 100 to 400 μg/mL (Table 1).

**Table 1:** Minimum inhibitory concentration of the Brazilian Red Propolis crude hydroalcoholic extract, and fractions against periodontopathogenic bacteria.

Minimum Inhibitory Concentration (µg/mL)					
Crude extract			Fractions		
Bacteria		Dichloromethane	Ethyl acetate	Hexane	*n*-Butanol
*Porphyromonas gingivalis* (ATCC 49417)	3.12	1.56	12.5	3.12	100
*Porphyromonas gingivalis* (clinical isolate)	12.5	25	400	100	–
*Fusobacterium nucleatum* (ATCC 10953)	100	200	400	–	–
*Fusobacterium nucleatum* (clinical isolate)	12.5	12.5	–	400	–
*Prevotella intermedia* (ATCC 15033)	6.25	6.25	200	200	200
*Prevotella intermedia* (clinical isolate)	50	100	–	400	–
*Actinomyces naeslundii* (ATCC 19039)	25	25	–	400	–
*Actinomyces naeslundii* (clinical isolate)	100	100	400	400	400

^a^Technique control strains: *Bacteroides fragilis* (ATCC 25285) and *Bacterioides thetaiotaomicron* (ATCC 29741)—Metronidazole: 1.47 and 2.95 µg/mL, respectively.—> 400 µg/mL was considered inactive.

**Table 2:** Minimum inhibitory concentration of the BRP isolated compounds against periodontopathogenic bacteria.

Minimum Inhibitory Concentration (µg/mL)						
Isolated compounds						
	Metilvestitol	Medicarpin	Vestitol	Neovestitol	Oblongifolin B	Guttiferone E
**Bacteria**						
*Porphyromonas gingivalis* (ATCC 49,417)	25	50	12.5	12.5	50	1.56
*Porphyromonas gingivalis* (clinical isolate)	100	200	100	100	6.25	6.25
*Fusobacterium nucleatum* (ATCC 10,953)	50	50	25	12.5	50	200
*Fusobacterium nucleatum* (clinical isolate)	–	50	100	50	3.12	3.12
*Prevotella intermedia* (ATCC 15,033)	–	100	100	50	6.25	12.5
*Prevotella intermedia* (clinical isolate)	–	–	200	100	50	50
*Actinomyces naeslundii* (ATCC 19,039)	400	100	200	50	6.25	6.25
*Actinomyces naeslundii* (clinical isolate)	–	–	200	100	25	50

^a^Technique control strains: *Bacteroides fragilis* (ATCC 25285)—Metronidazole: 1.47 µg/mL and *Bacteroides thetaiotaomicron* (ATCC 29741)—Metronidazole: 2.95 µg/mL. – > 400 µg/mL was considered inactive.

For the methylvestitol, the MIC values ranged from 25 to 400 μg/mL, medicarpin from 50 to 400 μg/mL, vestitol from 12.5 to 200 μg/mL, neovestitol from 12.5 to 100 μg/mL, oblongifolin B from 3.12 to 50 μg/mL, and guttiferone E from 1.56 to 200 μg/mL (Table 2).

### Antibiofilm activity of the BRP crude hydroalcoholic extract and isolated compounds

**Figure 2:**
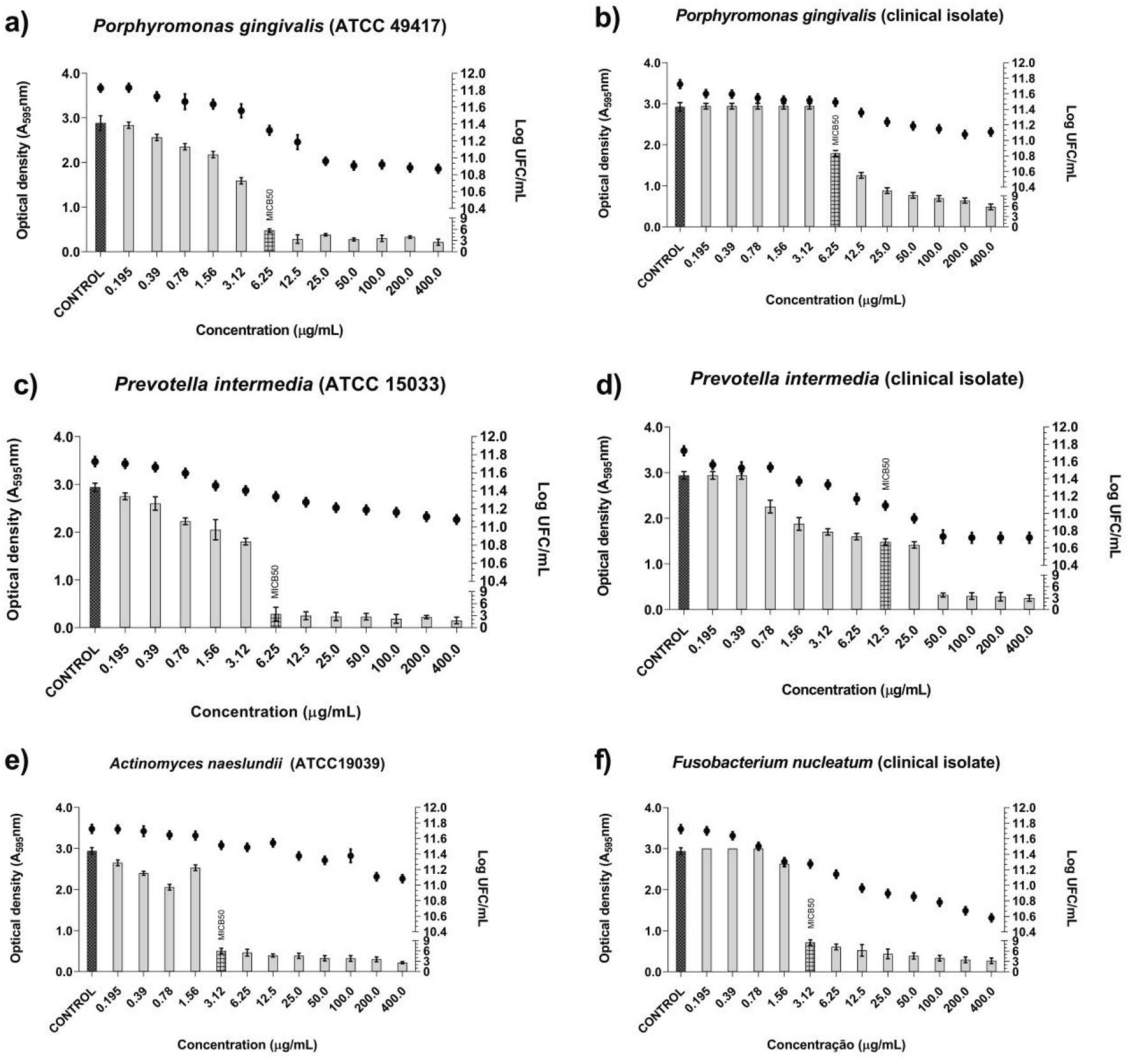
Antibiofilm activity of Brazilian Red Propolis crude hydroalcoholic extract samples and number of viable cells in monospecies biofilms formed by ATCC strains and clinical isolates included in the study**. **(**a**) *P. gingivalis* (ATCC 49417). (**b**) *P. gingivalis* (clinical isolate). (**c**) *P. intermedia* (ATCC 15033). (**d**) *P. intermedia* (clinical isolate). (**e**) *A. naeslundii* (ATCC 19039). (**f**) *F. nucleatum* (clinical isolate).

The BRP crude hydroalcoholic extract reduced the monospecies biofilm formation of the standard strains (ATCC) and their clinical isolates (Fig. 2). Additionally, the number of viable cells in the monospecies biofilm expressed as Log_10_ CFU/mL decreased (Fig. 2). The lowest MICB_50_ value obtained for the BRP crude hydroalcoholic extract against the monospecies biofilms was 3.12 μg/mL against *A. naeslundii* (ATCC 19039) and *F. nucleatum* (clinical isolate) (Fig. 2e and f). Against the other evaluated monospecies biofilms, the BRP crude hydroalcoholic extract presented MICB_50_ of 6.25 μg/mL, except for *P. intermedia* (clinical isolate), against which MICB_50_ was 12.5 μg/mL. However, even at concentrations above MICB_50_, we detected viable biofilm cells (Fig. 2a–f).

**Figure 3:**
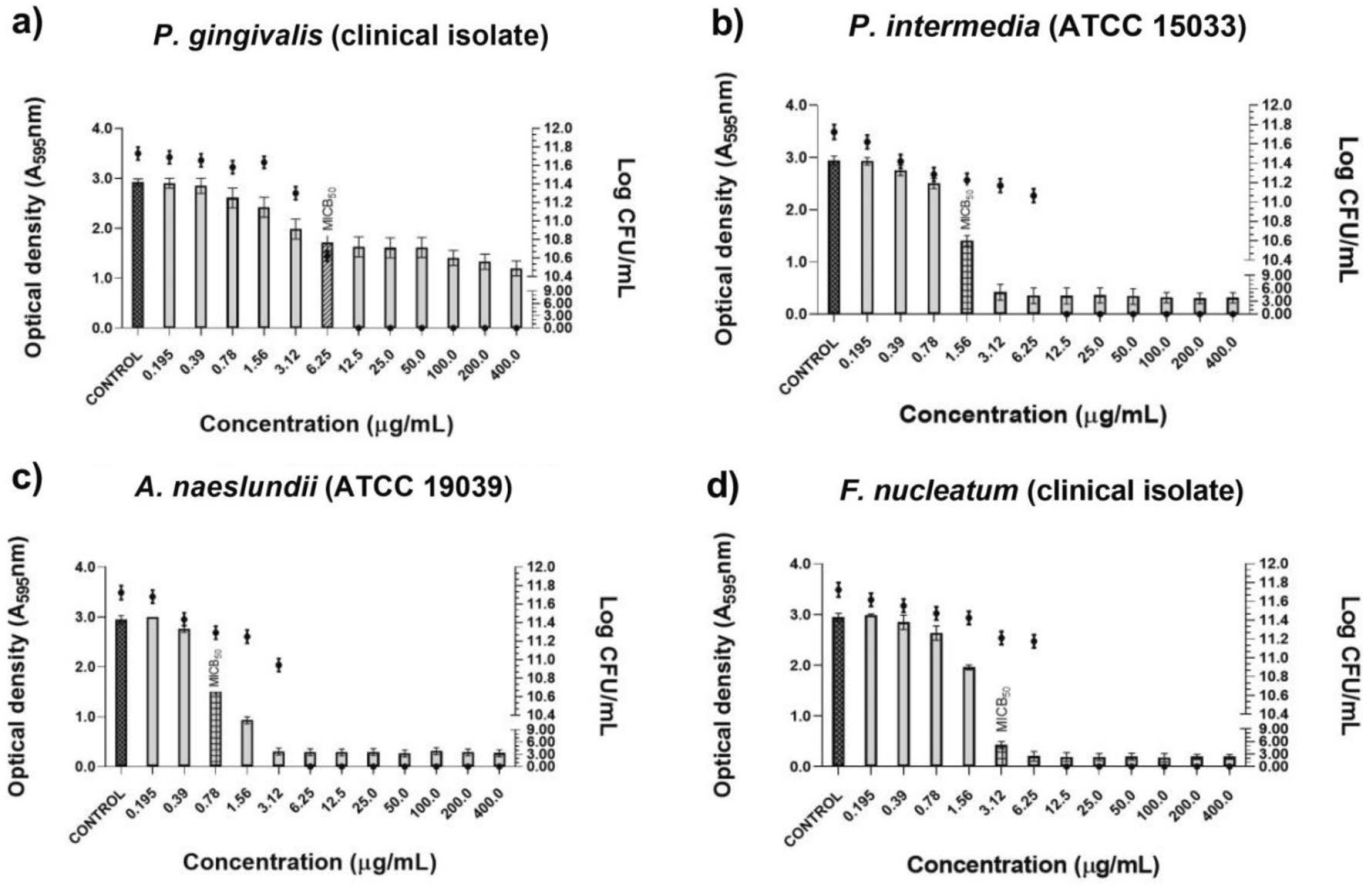
Antibiofilm activity of oblongifolin B and number of viable cells in monospecies biofilms formed by ATCC strains and clinical isolates included in the study. (**a**) *P. intermedia* (clinical isolate) (**b**) *P. intermedia* (ATCC 15033). (**c**) *A. naeslundii* (ATCC 19039). (**d**) *F. nucleatum* (clinical isolate).

As for the tested isolated compounds, they also reduced monospecies biofilm formation. In the presence of oblongifolin B (Fig. 3), the lowest MICB_50_ was 0.78 μg/mL against *A. naeslundii* (ATCC 19039) (Fig. 3c). Against the other evaluated monospecies biofilms, the MICB_50_ values ranged from 1.56 to 6.25 μg/mL. Oblongifolin B at 6.25 μg/mL eliminated *P. gingivalis* (clinical isolate) viable cells and, at 12.5 μg/mL, it eliminated *P. intermedia* (ATCC 15033) and *F. nucleatum* (clinical isolate) viable cells (Fig. 3a,b and d).

**Figure 4:**
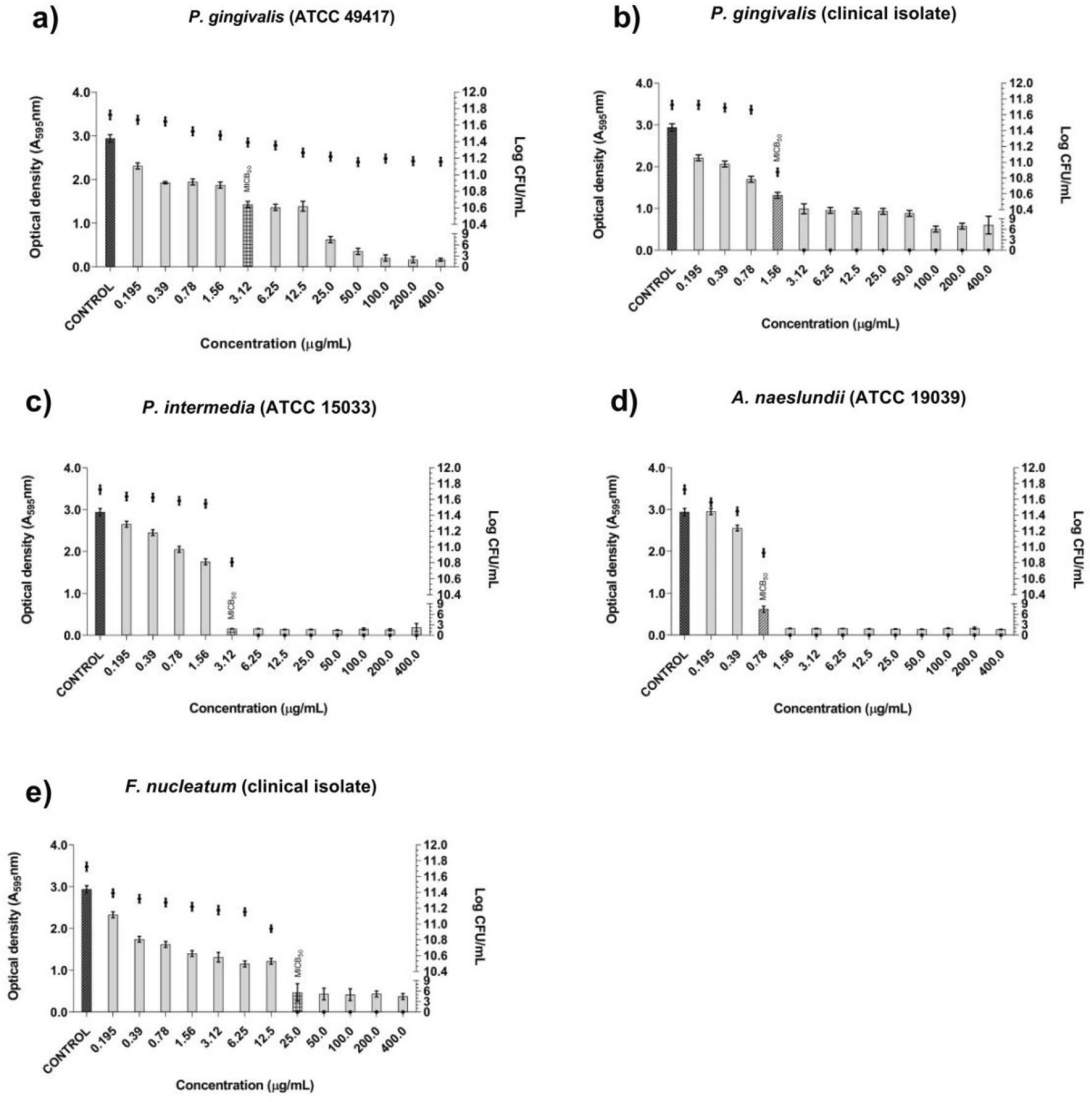
Antibiofilm activity of guttiferone E and number of viable cells in monospecies biofilms formed by ATCC strains and clinical isolates included in the study. (**a**) *P. gingivalis* (ATCC 49417). (**b**) *P. gingivalis* (clinical isolate). (**c**) *P. intermedia* (ATCC 15033). (**d**) *A. naeslundii* (ATCC 19039). (**e**) *F. nucleatum* (clinical isolate).

Guttiferone E presented low MICB_50_ (0.78 μg/mL) against *A. naeslundii* (ATCC 19,039) (Fig. 4d). Against the other evaluated monospecies biofilms, MICB_50_ ranged from 1.56 to 25 μg/mL (Fig. 4a,b,c and e). Guttiferone E eliminated all the biofilm cells from a concentration of 3.12 μg/mL against *P. gingivalis* (clinical isolate), 6.25 μg/mL against *P. intermedia* (ATCC 15033), 25 μg/mL against *F. nucleatum* (clinical isolate), and 1.56 μg/mL against *A. naeslundii* (ATCC 19039). As for *P. gingivalis* (ATCC 49417), we verified the presence of viable biofilm cells even at concentrations above MICB_50_ (Fig. 4a).

**Figure 5:**
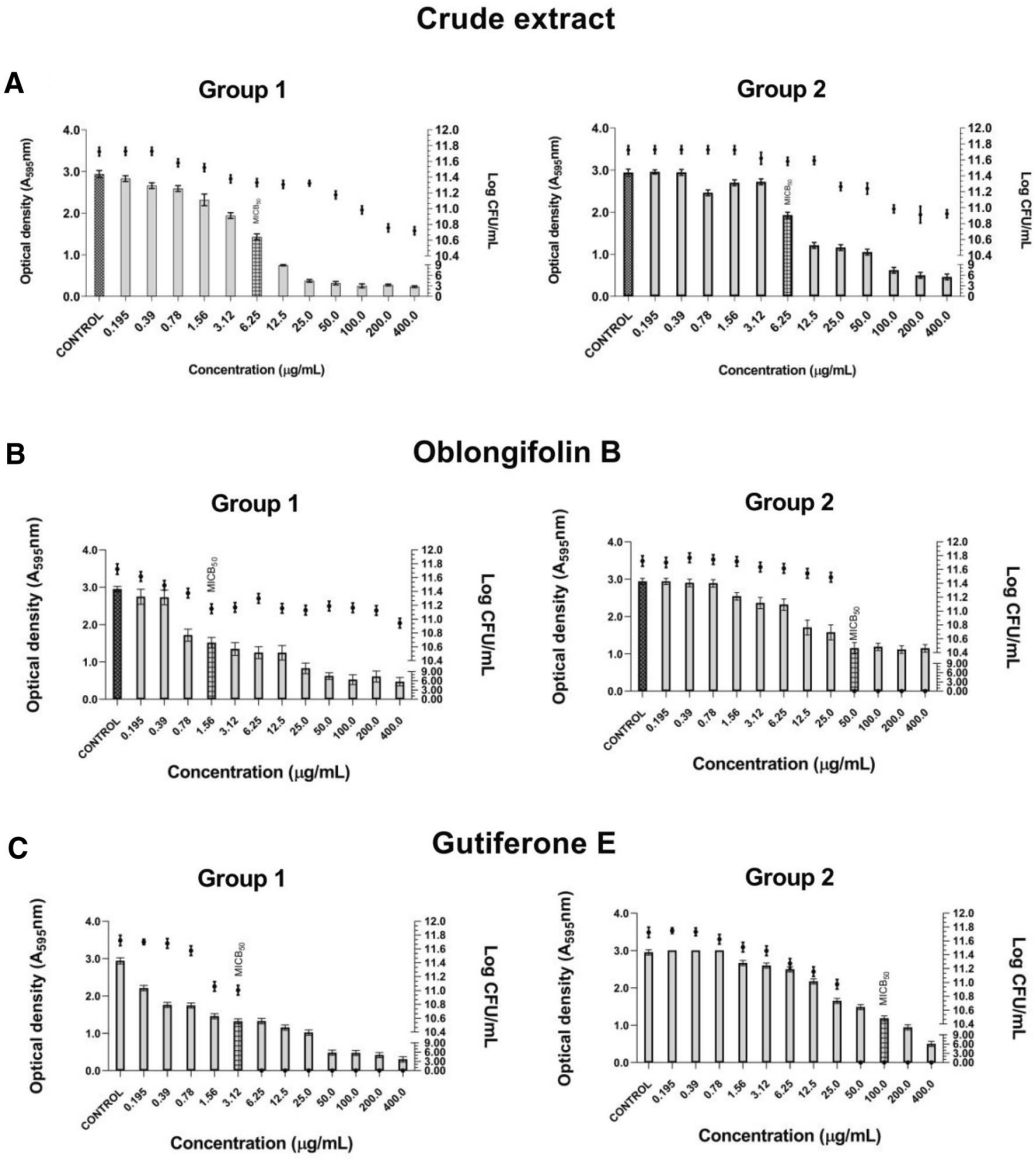
Antibiofilm activity of samples of Brazilian Red Propolis crude hydroalcoholic extract, oblongifolin B and guttiferone E and number of viable cells in multispecies biofilms formed by bacteria from groups 1 (standard strains) and 2 (clinical isolates). (**A**) Crude extract. (**B**) Oblongifolin B. (**C**) Guttiferone E.

We also assessed the activity of the BRP crude hydroalcoholic extract and isolated compounds against multispecies biofilm formed by standard strains (group 1) and clinical isolates (group 2) (Fig. 5). The BRP crude hydroalcoholic extract had MICB_50_ of 6.25 μg/mL against the group 1 multispecies biofilm However, even at higher concentrations, viable cells were still found in the biofilm. Similar results were found against the group 2 multispecies biofilm: MICB_50_ was 6.25 μg/mL, and there also were viable biofilm cells above the MICB_50_ concentration (Fig. 5A).

Concerning oblongifolin B, it had the lowest MICB_50_ against the group 1 multispecies biofilm (1.56 μg/mL); however, at concentrations above MICB_50_, cells remained viable in the biofilm. Against the group 2 multispecies biofilm, oblongifolin B presented MICB_50_ of 50 μg/mL and eliminated all the biofilm cells from the biofilm at this same concentration (Fig. 5B). On the other hand, guttiferone E showed MICB_50_ of 3.12 μg/mL against the group 1 multispecies biofilm, and 6.25 μg/mL guttiferone E eliminated all the cells from the biofilm. Against the group 2 multispecies biofilm, guttiferone E had higher MICB_50_ (100 μg/mL), but 50 μg/mL guttiferone E also eliminated all the viable cells from the biofilm (Figs. 5C).

Regarding the control (metronidazole), the MICB_50_ of monospecies biofilms ranged from 2.95 to 5.9 μg/mL. As for the mixed biofilms, the MICB_50_ was 2.95 μg/mL for both the biofilm formed by group 1 and the biofilm formed by group 2 (see supplementary material Figures S2 and S3).

### Effects of the BRP crude hydroalcoholic extract and isolated compounds on CHIKV replication

**Figure 6:**
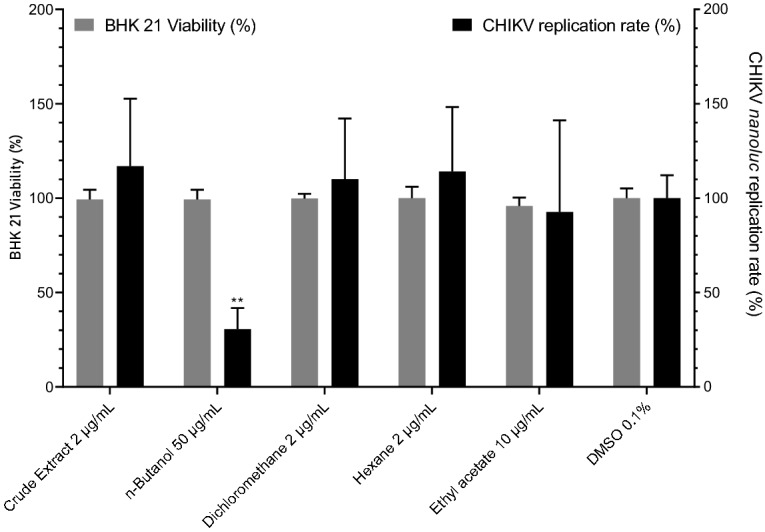
Cell viability and CHIKV replication rates in the presence of Brazilian Red Propolis crude hydroalcoholic extract and fractions.

To further evaluate the effects of BRP extract and its isolated compounds, BHK 21 cells were treated with each extract at 50, 10 and 2 μg/mL and cell viability was measured 16 h later. The results demonstrated that cells tolerated *n*-Butanol at 50 μg/mL (98.4%), ethyl acetate at 10 μg/mL (95.9%), while the crude extract, dichloromethane, and hexane at 2 μg/mL (99.3, 99.8, and 100%, respectively), (Table 3). Through the employment of BHK-21 cells infected with CHIKV-*nanoluc*, the anti-CHIKV activities of each sample were evaluated, at the maximum non-cytotoxic concentrations selected through the viability assay. The results demonstrated that *n*- Butanol significantly inhibited 69% of CHIKV replication (Fig. 6). The other samples presented no effect on CHIKV infection (Fig. 6).

**Table 3:** Cell viability percentage in the presence of the BRP crude hydroalcoholic extract or fractions at 50, 10, and 2 µg/mL.

Sample/concentration	50 µg/mL	10 µg/mL	2 µg/mL
Crude extract	57.3	83.2	99.3
*n*- Butanol	98.4	100.3	100.6
Dichloromethane	48.3	85.1	99.8
Hexane	45.7	83.3	100.0
Ethyl acetate	66.4	95.9	96.4

**Figure 7:**
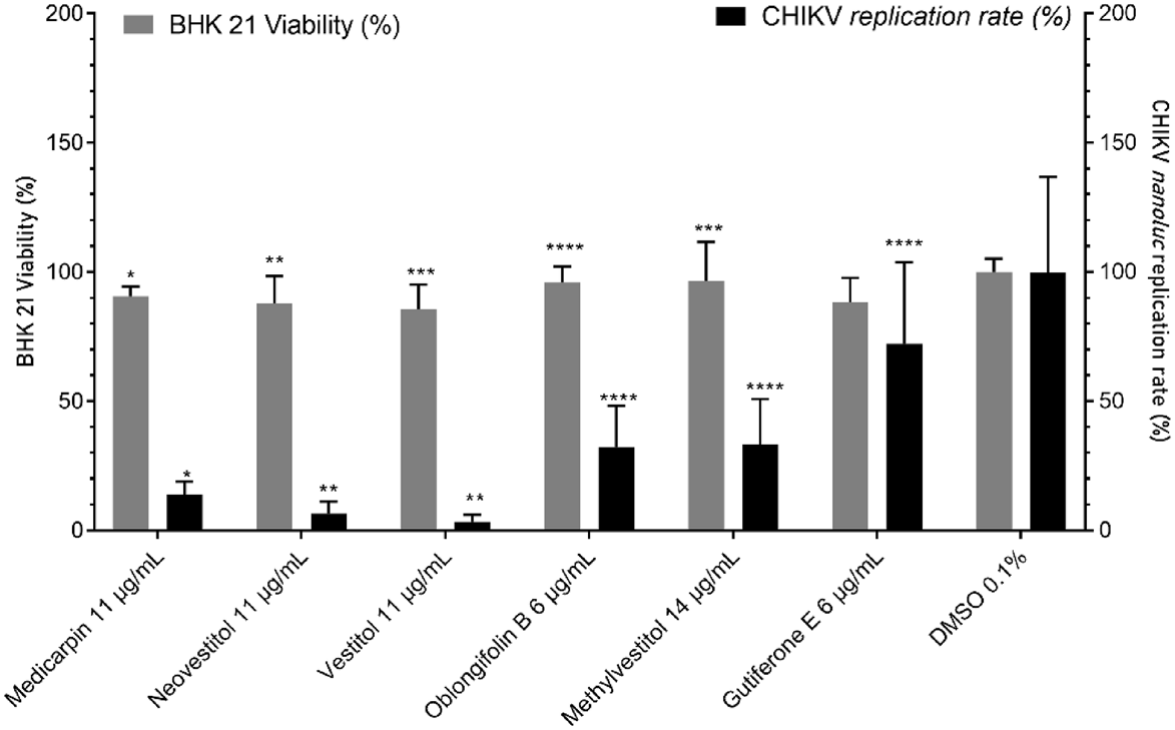
Effects of isolated compounds at on CHIKV infection and cell viability.

For the isolated substances (medicarpin, neovestitol, vestitol, oblongifolin B, methylvestitol and, guttiferone E), BHK-21 cells were treated with concentrations of each compound ranging from 32 to 0.5 μg/mL. As an outcome, the treatment with compounds in concentrations over 3 µg/mL presented cell viability rates higher than 80% (Table 4), and the highest non-cytotoxic concentration of each compound was selected for the antiviral assay. Since medicarpin, neovestitol and vestitol at 14 µg/mL presented cytotoxicity (Table 4), and at 3 µg/mL showed no antiviral activity (Supplementary Figure S4), the alternative concentration of 11 µg/mL was selected to the further assays. Therefore, the antiviral activity of medicarpin, neovestitol and vestitol was tested at 11 µg/mL, guttiferone E and oblongifilin B at 6 µg/mL, and methylvestitol at 14 µg/mL. The compounds medicarpin, neovestitol and vestitol inhibited CHIKV replication in vitro in 86%, 94%, and 97% respectively (Fig. 7).

**Table 4:** BHK-21 cell viability in the presence of the BRP isolated substances at concentrations ranging from 32 to 0.5 μg/mL.

Sample	Concentration (µg/mL)	Cell viability (%)
Medicarpin	14	84
	3	122
	0.5	113
Neovestitol	14	66
	3	110
	0.5	120
Vestitol	14	74
	3	117
	0.5	125
Oblongifolin B	32	72
	6	98
	1	99
Methylvestitol	14	108
	3	103
	0.5	103
Guttiferone E	32	15
	6	88
	1	90

### Toxicity assessment in *Caenorhabditis elegans*

**Figure 8:**
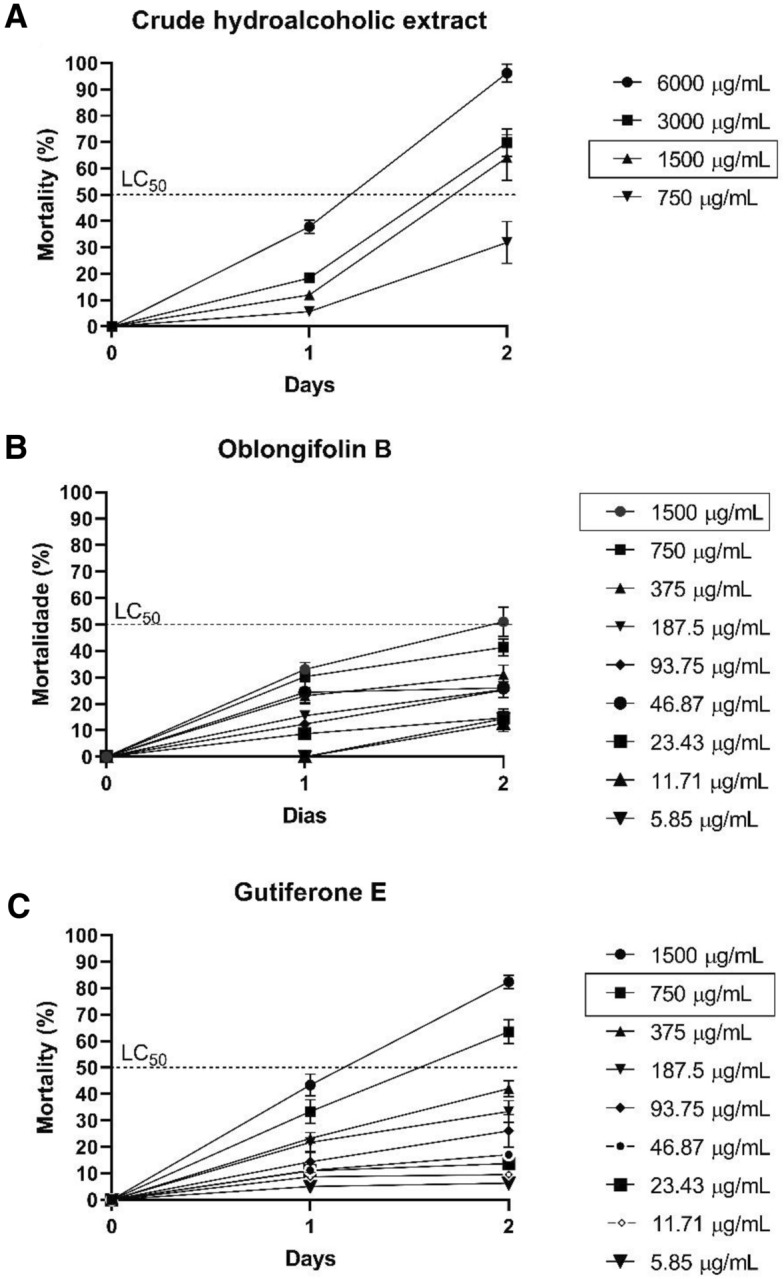
Evaluation of the toxicity of the Brazilian Red Propolis crude hydroalcoholic extract and isolated compounds guttiferone E and oblongifolin B in the *C. elegans *in vivo model. (**A**) Crude hydroalcoholic extract. (**B**) Oblongifolin B. (**C**) Guttiferone E.

To assess the toxicity of the BRP crude hydroacoholic extract and isolated compounds in an in vivo system, the technique for determining the lowest concentration capable of killing 50% (LC_50_) of the larvae in relation to the incubation time was employed. Figure 8 shows the toxicity evaluation of the BRP crude hydroalcoholic extract, oblongifolin B, and guttiferone E as a function of time and concentration. The LC_50_ of the BRP crude hydroalcoholic extract and oblongifolin B was 1500 μg/mL, determined on the second day of incubation (Fig. 8A and B). On the other hand, guttiferone E had LC_50_ of 750 μg/mL, determined on the last day of incubation (Fig. 8C).

## Discussion

For years, propolis has been used to treat infections in folk medicine, and its antimicrobial potential has been demonstrated by the scientific community^15^. This biological potential can be related to its differentiated chemical composition.

Sesquiterpenes, pterocarpans, and isoflavans characterize Brazilian red propolis. Red propolis chemical composition is much different from other propolis types, such as brown propolis, which is characterized by hydrocarbons, aldehydes, and monoterpenes; and green propolis, which is characterized by polycyclic aromatic hydrocarbons, sesquiterpenes, and naphthalene derivatives^31^.

Vestitol, neovestitol, and medicarpin have been reported as major compounds in red propolis From Canavieiras, Bahia State, Brazil. On the other hand, formononetin, calycosin, biochanin A, and isoliquiritigenin were detected at lower concentrations^17^. Guttiferone E and oblongifolin B were described as chemical markers of red propolis^14^, but they appear to be at lower concentrations in the studied sample compared to the isoflavans. The triterpenes *β*-amyrin and glutinol have also been described in BRP from this location^14^.

According to Rios and Recio^32^ and Gibbons^33^, MIC values below 100 µg/mL for crude hydroalcoholic extract or below 10 µg/mL for isolated compounds are considered promising when evaluating the antibacterial activity of plant extracts, essential oils, and compounds isolated from natural sources. On the basis of these criteria and considering the MIC values presented here for all the evaluated BRP samples, the BRP crude hydroalcoholic extract and the isolated compounds guttiferone E and oblongifolin B displayed the best inhibition activity against most of the evaluated bacteria.

The red propolis dichloromethane fraction was not tested since the selection was based on the effect of the individual constituents of each fraction. The main compounds of hexane fraction, oblongifolin B and guttiferone E, displayed good activity at the individual testing, compared with the dichloromethane fraction individual compounds.

These samples showed antibacterial activity mainly against *P. gingivalis* (ATCC 49417), considered the most clinically important species in the development of periodontal disease^34^ and *F. nucleatum* (clinical isolate) bacteria, also considered a relevant pathogen since it worsens gingival inflammation and tooth loss^35^. These results demonstrated the relevance of these natural products in periodontal disease control and treatment. In this paper, the BRP crude hydroalcoholic extract, fractions (*n*-hexane, dichloromethane, ethyl acetate, and *n*-butanol), and isolated compounds (methylvestitol, medicarpin, vestitol, neovestitol, oblongifolin B, and guttiferone E) were analyzed for their antibacterial activity against clinical isolates and the ATCC strains. The ATCC strains are more stable from a genetic viewpoint and would thus represent the bacterium species, thereby enabling comparison with other investigations. The in vitro assay furnishes a reliable indication of how the microorganism responds to the target agent, and extrapolation of the results for that species or even genus should be accepted. Clinical isolates (also known as wild strains) are bacteria that can have their metabolism altered by environmental conditions and their genetics modified by circulation in the population, which would justify the relevance of evaluating these two types of strains.

The MIC values (1.56–400 µg/mL) for the other evaluated bacteria were significantly lower as compared to literature data. Bueno-Silva et al.^29^ evaluated the antibacterial activity of the crude extract and isolated compounds neovestitol and vestitol obtained from BRP from the same botanical origin against *A. naeslundii* (ATCC 12104), and they reported MIC values of 25, 25, and 50 µg/mL, respectively. Here, neovestitol and vestitol were not promising against several of the evaluated periodontal bacteria. Another point to emphasize is that the MIC value for the crude extract reported by Bueno-Silva et al.^29^ against *A. naeslundii* (ATCC 12104) resembled the value we obtained against *A. naseslundii* (ATCC 19039), suggesting a species susceptibility relation for the crude extract.

Santos et al.^36^ evaluated the antibacterial activity of the aqueous hydroalcoholic extract and fractions (hexane, dichloromethane, and ethyl acetate) obtained from a different type of propolis, from the region of “Cachoeira da Prata”, Minas Gerais—Brazil, which is also collected from *Apis mellifera* bees. The tested bacteria were periodontal *F. nucleatum* (ATCC 10953), *P. gingivalis* (ATCC 33277), and *P. intermedia* (ATCC 25611). The extract gave MIC values of 1024, 256, and 256 µg/mL against *F. nucleatum* (ATCC 10953), *P. gingivalis* (ATCC 33277), and *P. intermedia* (ATCC 25611), respectively. As for the fractions, the MIC values ranged from 512 to > 1024 µg/mL. The MIC results against these bacteria were higher than those presented here for the BRP crude hydroalcoholic extract and fractions against the same bacterial species but from different strains: we found MIC values of 100, 3.12, and 6.25 µg/mL for the crude extract against *F. nucleatum* (ATCC 10953), *P. gingivalis* (ATCC 49417), and *P. intermedia* (ATCC 15033), respectively. As for the fractions, we found MIC values ranging from 12 to 400 µg/mL against the same bacteria. Therefore, compared to the results described by these authors, the BRP crude hydroalcoholic extract and fractions employed here were more effective against periodontal bacteria.

Additionally, these authors compared the MIC results they obtained with the crude extract and fractions by ANOVA analysis. They did not find any differences in the antibacterial activity of the fractions or extract against the evaluated bacteria. This corroborates our results: the BRP crude hydroalcoholic extract was more promising than the fractions and gave better values against all the evaluated bacteria, while the fractions presented antibacterial activity against only two bacteria (*P. gingivalis* ATCC 49417 and clinical isolate).

Shabbir et al.^37^ evaluated the activity of the crude propolis extract collected in Skardu (Pakistan) originating from *Robinia pseudoacacia*, *Elegnus agustifolia* (Russian olive), and *Acacia modesta*, collected from *Apis mellifera* bees. The natural products afforded MIC values ranging from 64 to 512 µg/mL against *P. gingivalis* and *P. intermedia* clinical isolates. Our results suggested that BRP is more effective than the propolis used by Shabbir et al.^37^ since the MIC values we obtained for the BRP crude hydroalcoholic extract against *P. gingivalis* and *P. intermedia* clinical isolates were lower (12.5 and 6.25 µg/mL, respectively). Therefore, BRP proved to have more promising activity than propolis from other countries given that it is composed of unique compounds that do not normally occur in other types of propolis^15^.

Inhibiting biofilm formation by these bacteria can contribute to reducing periodontitis. Indeed, Al-Ahmad et al.^38^ described that *A. naeslundii* and *F. nucleatum* play the roles of initial colonizing bacterium and late colonizer, respectively. The latter bacterium was shown to be present at rates greater than 50% after 62 h of biofilm formation, contributing to increased inflammation and tooth loss^38^.

Other studies have evaluated the BRP monospecies antibiofilm activity against other types of bacteria. de Souza Silva et al.^39^ evaluated the antibiofilm activity of the BRP crude hydroalcoholic extract (BRP collected in the same region as the BRP used in our study) coated on polymeric nanoparticles against *Staphylococcus aureus* (ATCC 25923), *Staphylococcus aureus* (ATCC 33591), *Staphylococcus aureus* (ATCC 43300), and *Pseudomonas aeruginosa* (ATCC 27853). The free BRP crude hydroalcoholic extract and the extract coated on nanoparticles inhibited the biofilm formed by the Gram-positive strains more effectively as compared to the biofilm formed by the Gram-negative strains, with biofilm inhibitory concentration values ranging from 15.6 to 125 μg/mL against the *S. aureus* strains and from 100 to 1560 μg/mL against the *P. aeruginosa* strain. These results corroborated our findings given that we verified the lowest MICB_50_ values against the Gram-positive bacterium *A. naeslundii* (ATCC 19039) 3.12 μg/mL for the crude extract and 0.78 μg/mL for the isolated compounds oblongifolin B and guttiferone E.

Miranda et al.^13^ evaluated the antibiofilm activity of the crude hydroalcoholic extract of BRP from the same botanical origin as the BRP used here. These authors divided the evaluated bacteria into complexes (*Actinomyces*, purple, yellow, green, orange, red, and others). At extract concentrations of 800 and 1,600 μg/mL, the authors showed a 40 and 45% decrease in the metabolic activity of the multispecies biofilms formed by these complexes, respectively. de Figueiredo et al.^30^ also evaluated the antibiofilm activity of the BRP crude extract at 1600, 800, and 400 μg/mL and obtained 56, 56, and 57% reduction in the biofilm metabolic activity, respectively. Our study did not assess eradication, but it evaluated the ability of the BRP samples to inhibit biofilm formation. According to Wei et al.^40^ inhibiting biofilm formation is much more important than erradicating it because biofilm formation inhibition prevents bacterial growth and, hence, bacterial maturation. The results presented here demonstrated that the BRP samples inhibited multispecies biofilm formation by periodontal bacteria by at least 50%. Oblongifolin B gave the lowest MICB_50_ value (1.56 μg/mL) against the multispecies biofilm formed by the ATCC strains. As for the multispecies biofilm formed by the clinical isolates, the lowest MICB_50_ value was 6.25 μg/mL. These results suggested that the isolated compounds oblongifolin B and guttiferone E inhibited all the viable cells of most monospecies and multispecies biofilms formed by the bacteria included in this study. This pointed out that the BRP samples can inhibit biofilm formation and reach the cells within this bacterial community, eliminating them and leaving only the glycoprotein conjugate without live cells^13^.

It is worth mentioning that the MICB_50_ values found in this paper are relatively low, especially for clinical isolates that are generally more resistant and demand higher concentrations. However, at the lowest concentration capable of inhibiting biofilm formation by at least 50%, the so-called MICB_50_, we demonstrated an inhibition of at least 50% of the biofilm. In other words, this did not correspond to total inhibition of the biofilm, which would probably require a higher concentration.

Propolis bioactive components such as flavonoids, esters, alcohols, essential oils, and other organic compounds have already been demonstrated to display antiviral activity against viruses such as herpes viruses (HSV-1 and HSV-2), sindbis virus, parainfluenza virus, cytomegalovirus, HIV, and *Varicella zoster* (HSV-1 and HSV-2), sindbis virus, parainfluenza virus, cytomegalovirus, HIV, and *Varicella zoster*^41,42^. In addition to the BRP antibacterial and antibiofilm activities demonstrated in this study, we evaluated the anti-CHIKV activity of the BRP crude hydroalcoholic extract, fractions, and pure substances. We assessed the BHK-21 cell viability in the presence of the BRP samples by the MTT assay. In drug discovery, samples are considered non-toxic when the cell viability rate is above 50%, moderately cytotoxic when the cell viability rate varies between 25 and 50%, and highly cytotoxic when the cell viability rate is less than 25%^43^. In this study, all samples evaluated showed cell viability equal to or higher than 80% at a concentration of 50 μg/mL for the crude extract and fractions and 3 μg/mL for isolated compounds (Tables 3 and 4). All the BRP samples evaluated in this study provided BHK-21 cell viability equal to or higher than 80% (Tables 3 and 4), which was higher than the cell viability found by Rufatto et al. ^20^, (between 14.5 and 46%).

Regarding the infection assays, the isolated compounds neovestitol and vestitol furnished the most promising results, with virus infection inhibition rates of 94 and 97%, respectively (Fig. 7). Even though several natural compounds with antiviral activity, including anti-CHIKV activity, have been described, neovestitol and vestitol have not had their antiviral potential screened. Our results showed higher inhibition rates than those reported in other studies with natural molecules, such as the study of Pohjala et al.^44^, who obtained an infection inhibition limit of at most 75% when they screened 356 compounds, being 123 of them natural compounds. To the best of our knowledge, there are no reports on the anti-CHIKV activity of BRP or isolated compounds. This shows the importance of capitalizing the BRP potential as candidate for antiviral treatment. Our study has pioneered evaluation of the BRP anti-CHIKV activity and has achieved expressive inhibition rates, paving the way for the development of antivirals against CHIKV as well as other viruses.

For BRP to be safely applicable, its toxicity must be evaluated in different experimental models. The murine model is the most used in vivo model to assess the toxicity of treatments, but it has disadvantages such as high cost, difficult maintenance, and delay in obtaining results, among others^45^. Therefore, here we evaluated toxicity by using another in vivo model, the nematode *C. elegans*, a complete animal with digestive, reproductive, endocrine, and neuromuscular systems. Apart from being small, having a short life cycle, and being easy to maintain, *C. elegans* possesses 60–80% genetic homology with humans^46^. In this context, we evaluated the most promising BRP samples for their toxicity against *C. elegans*. The lowest concentration capable of killing at least 50% of the larvae (LC_50_) was 1500 μg/mL for the BRP crude hydroalcoholic extract and oblongifolin B and 750 μg /mL for gutiferon E. These values were significantly higher than all the MIC and MICB_50_ concentrations reported in this study.

Moreover, below this concentration, even after the larvae had been exposed to BRP samples for two days, LC_50_ was not reached, demonstrating the non-toxic profile of these natural products. Interistingly, the LC_50_ values of the BRP samples against *C. elegans* obtained in this study were higher than the LC_50_ values of other types of Brazilian propolis evaluated against *C. elegans*. For example, Campos and collaborators (2015)^47^ reported that propolis samples possessed LC_50_ of 461.8 μg/mL. Here, the BRP concentrations determined as toxic were high, above the highest MIC value (400 μg /mL). Therefore, propolis is not toxic at the concentrations used in this study and can be safely employed at concentrations below 1500 and 750 μg /mL. The results obtained here are extremely relevant, because through different methodologies the antibacterial activity of Brazilian red propolis was demonstrated against a panel of periodontopathogenic bacteria. Another point to highlight is the anti-CHIKV activity of the BRP isolated compounds. *Chikungunya* infection has a high incidence and severity, therefore, the search for new treatment options is highly desirable. Our results constitute an initial step for further studies of BRP as an alternative approach for treating various infectious diseases.

## Conclusion

The Brazilian red propolis used in this study has antibacterial activity against a panel of periodontopathogenic bacteria. Furthermore, it’s crude extract and isolated compounds oblongifolin B and guttiferone E at concentrations similar to or slightly above the MIC concentrations inhibits monospecies and multispecies biofilms by over 50%. Medicarpin, neovestitol, and vestitol strongly inhibit CHIKV infection in vitro. Besides, toxicity tests on *C. elegans* demonstrated that the crude extract, oblongifolin B, and guttiferone E are non-toxic, proving to be safe and promising so that in the future, these samples of propolis can be used as medicine.

## Methods

### Crude hydroalcoholic extract, fractions, and isolated compounds

BRP was collected in Canavieiras Bahia State, Brazil, in March 2019 at the Canavieiras Beekeepers Association (COAPER). BRP was frozen and extracted with 70% hydroalcoholic ethanol solution, as described by Santiago et al.^48^. The BRP crude hydroalcoholic extract was partitioned with organic solvents (hexane*,* dichloromethane, ethyl acetate, and *n-*butanol). Authentic standards from BRP (7-*O*-methylvestitol, medicarpin, vestitol, neovestitol, oblongifolin B, and guttiferone E) previously isolated by our research group were used to characterize the samples^17^.

Chromatographic analysis of BRP extract and its fractions were performed on a Waters 2695 HPLC instrument, coupled to a 2998 photodiode array detector (PDA), with Empower 3 software as a controller. Chromatographic profiles were carried out on a Supelco Ascentis Express C-18 (150 × 4.6 mm, 2.7 µm) column. Mobile phase with water (A) (0.1% formic acid) and acetonitrile (B) was used as follows: 10 → 100% of B until 80 min; 100% of B in 89 min; 100 → 10% in 90 min, maintaining the condition until 95 min. The injections were performed on a flow rate of 1 mL/min, a 40 °C, and an injection volume of 10 µL. Chromatograms were recorded at 275 nm.

For the antibacterial, antiviral, and toxicicity assays were used the crude hydroalcoholic extract of BRP, fractions in dichloromethane, hexane, ethyl acetate, *n*-butanol, as well as the isolated compounds guttiferone E, oblongifolin B, methylvestitol, medicarpin, vestitol, and neovestitol.

### Bacterial strains, *Chikungunya* virus and animal model employed in the study

The periodontopathogenic bacterial strains employed in the antibacterial and antibiofilm activity assays were obtained from the *American Type Culture Collection* (ATCC); their respective clinical isolates were obtained from human periodontal infections. The strains included *Porphyromonas gingivalis* (ATCC 49417 and clinical isolate), *Fusobacterium nucleatum* (ATCC 10953 and clinical isolate), *Prevotella intermedia* (ATCC 15033 and clinical isolate), and *Actinomyces naeslundii* (ATCC 19039 and clinical isolate). These bacteria are part of the collection of the Antimicrobial Assays Laboratory (LEA, abbreviation in Portuguese) of the Federal University of Uberlândia (UFU) and were cryopreserved at − 20 °C. For the in vivo toxicity assays, the mutant strain *Caenorhabditis elegans* AU37, obtained from the Genetics Center (CGC, University of Minnesota), was used.

For the antiviral assays, a CHIKV expressing the *Nanoluciferase* reporter (CHIKV-*nanoluc*) based on the CHIKV LR2006PYY1 strain (East/Central/South African genotype) was rescued^49^. The protocols were carried out as described previously^50^.

### Determination of the minimum inhibitory concentration^51^

The antibacterial activity of the BRP crude hydroalcoholic extract, fractions, and isolated compounds were evaluated by the broth microdilution method, in triplicate. The assays were conducted in 96-well microplates; the methodology recommended by the Clinical and Laboratory Standards Institute^52^, with modifications, was followed. The inoculum was standardized to the McFarland 0.5 scale and diluted to a bacterial concentration of 1.5 × 10^6^ CFU/mL in the wells. To prepare the samples, the BRP crude hydroalcoholic extract, fractions, or isolated compounds were solubilized in 5% dimethyl sulfoxide (DMSO) and diluted in Brucella broth supplemented with hemin (5.0 mg/mL) and menadione (1.0 mg/mL); a twofold serial dilution with concentrations ranging from 0.195 to 400 µg/mL was used. Control of 5% DMSO was performed, and the solvent did not interfere with bacterial growth at this concentration. It was also performed the following controls: inoculum (all the bacteria used in the test + the culture medium), to observe the viability of the bacteria; broth, to guarantee that the culture medium is sterile; and BRP sample, to guarantee that this solution is sterile. The microplates were incubated in an anaerobic chamber (Don WhitleyScientific, Bradford, U.K.) under anaerobic conditions (80% N_2_, 10% CO_2_, and 10% H_2_) at 37 °C for 72 h. Rezasurin was used to reveal bacterial growth—the blue color indicated absence of bacterial growth, and the pink color indicated presence of bacteria^53^. As a control technique, metronidazole from 0.0115 to 5.9 μg/mL was used against the control bacteria *Bacteroides fragilis* (ATCC 25285) and *Bacteroides thetaiotaomicron* (ATCC 29741)^52^.

### Evaluation of antibiofilm activity monospecies and multispecies by Minimum Inhibitory Concentration of Biofilm (MICB_50_)

To assess the antibiofilm activity, the BRP samples that presented the most promising MIC results against four or more bacteria were submitted to the Minimum Inhibitory Concentration of Biofilm (MICB_50_) assay. MICB_50_ is defined as the lowest concentration of the microbial agent that can inhibit biofilm formation by at least 50%^40^ and is calculated using the following equation:

$$1 - \frac{{\left( {{\text{Absorbance }}\left( {595{\text{nm}}} \right){\text{of the well containing the treated sample }}} \right)}}{{{\text{Absorbance }}\left( {595{\text{nm}}} \right){\text{ of the untreated control well }}}} \times 100$$

Here, MICB_50_ was determined as described in the CLSI guidelines (2007)^52^, with modifications. First, the capacity of the analyzed strains to grow in the sessile mode was verified. All the strains at 1.5 × 10^6^ CFU/mL formed monospecies and multispecies biofilms after incubation at 37 °C for 72 h (data not shown).

For the monospecies biofilms, 100 μL of each bacterium inoculum at 1.5 × 10^6^ CFU/mL was added to the well with the propolis samples to be evaluated at concentrations from 0.195 to 400 µg/mL (crude hydroalcoholic extract, oblongifolin B and guttiferone E). The microplates were incubated in an anaerobic chamber at 37 °C for 72 h. For the multispecies biofilms, the main periodontopathogenic bacteria found in the oral biofilm were selected and divided into two groups: group 1 consisted only of the standard bacteria (*P. gingivalis* ATCC 49417, *P. intermedia* ATCC 15033, and *A. naeslundii* ATCC 19039), while group 2 was composed only by the *P. gingivalis*, *P. intermedia*, and *F. nucleatum* clinical isolates. The antibiofilm activity of the most promising BRP samples was evaluated against the multispecies biofilm formed by group 1 bacteria and against the multispecies biofilm composed by group 2 bacteria. For this purpose, 33.33 μL of each evaluated bacterium, totaling 100 μL of bacterial inoculum, at 1.5 × 10^6^ CFU/mL was added to the wells with the propolis samples to be evaluated at concentrations from 0.195 to 400 µg/mL (crude hydroalcoholic extract, oblongifolin B and guttiferone E). The microplates were incubated under the same conditions as the monospecies biofilm microplates. The standard antibiotic metronidazole was used as a control at concentrations from 0.0115 to 5.9 μg/mL with MIC_50_ (see supplementary material, Figures S2 and S3). Control of 5% DMSO was performed, and the solvent did not interfere with bacterial growth at this concentration. It was also performed the following controls: inoculum (all the bacteria used in the test + the culture medium), to observe the viability of the bacteria; broth, to guarantee that the culture medium is sterile; and BRP sample, to guarantee that this solution is sterile. After incubation, the supernatant culture was withdrawn, and the planktonic cells were removed by washing the wells with ultrapure distilled water. Monospecies and multispecies biofilms were fixed with methanol and stained with 2% crystal violet^54^. The reading was performed in a microplate reader (GloMax®) at 595 nm. Reading was performed in a microplate reader (GloMax®) at 595 nm. The experiments were carried out in triplicate and independent events.

### Evaluation of the inhibition of biofilm formation by counting microorganism

This assay was performed for monospecies and multispecies biofilms according to de Souza Silva et al.^39^, as described below. Two microplates were incubated, one for MICB_50_ determination, and the other for microorganism count. After the microorganism count microplate was incubated, the supernatant was withdrawn, and the planktonic cells were removed by washing the wells with ultrapure distilled water. Subsequently, supplemented Brucella broth was added to all the microplate wells, and the biofilm was detached from the well after an ultrasound bath. Then, tenfold serial dilutions were performed in each well of a 96-well microplate, and 50 μL of each well, corresponding to each dilution avaliated was placed on two plates of Brucella agar supplemented with horse blood (5%), hemin (5.0 mg/mL), and menadione (1.0 mg/mL). Each of the plates were fractionated into eight parts, as described by Harrison et al.^55^ and incubated in an anaerobic chamber for 37 °C. After 72 h, the Colony Forming Units (CFU) count was performed in each plate. The results were expressed as Log_10_ (CFU/mL), and the assays were independently performed in triplicate.

### Mammalian cells for antiviral assays

The BHK-21 cells (fibroblasts derived from Syrian golden hamster kidney; ATCC CCL-10) were maintained in Dulbecco’s modified Eagle’s medium (DMEM, Sigma-Aldrich) supplemented with 100 U/mL penicillin (Hyclone Laboratories), 100 mg/mL streptomycin (Hyclone Laboratories), 1% dilution of stock of non-essential amino acids (Hyclone Laboratories), and 1% fetal bovine serum (FBS, Hyclonen Laboratoires) in a humidified 5% CO_2_ incubator at 37 °C.

### Cell viability through MTT for antiviral assays

BHK-21 cell viability in the presence of the tested BRP samples was measured by the MTT [3-(4,5-dimethylthiazol-2-yl)-2,5-diphenyl tetrazolium bromide] (Sigma–Aldrich) assay. BHK-21 cells were cultured in 48-well microplates and treated with different concentrations of the tested BRP sample at 37 °C for 16 h. Then, media containing the tested BRP sample was removed from the 48-well microplate. Next, 1 mg/mL MTT solution was added to each well, incubated for 30 min, and replaced with 300 μL of DMSO to solubilize the formazan crystals. Absorbance was measured at 490 nm on a Glomax microplate reader (Promega). Cell viability was calculated according to the equation (T/C) × 100%, where T and C represent the optical density of the treated well and control groups, respectively. DMSO was used as untreated control^50^.

### Antiviral activity against CHIKV infection in vitro

For initial screening of the anti-CHIKV activity of the BRP crude hydroalcoholic extract and isolated compounds, HK-21 cells were seeded at a density of 5 × 10^4^ cells per well in 48-well microplates 24 h before the infection. CHIKV-*nanoluc* at a multiplicity of infection^56^ of 0.1 and the tested isolated compound or extract were simultaneously added to the cells. The cells were harvested in Renilla luciferase lysis buffer (Promega) 16 h post-infection (h.p.i.), and virus replication was quantified by measuring nanoluciferase activity with the Renilla luciferase Assay System (Promega). The CHIKV replication rates were calculated according to the equation (T/C) × 100%, where T and C represent the optical density of the treated well and control groups, respectively. DMSO 0.1% was used as untreated control.

### Toxicity assessment in *Caenorhabditis elegans*

Toxicity evaluation was performed for the most promising BRP samples in the CIM, using the in vivo model of *C. elegans*, according to Andrade et al.^57^ and Singulani et al.^58^. The *C. elegans* AU37 mutant strain was cultivated in Nematode Growth Medium (NGM) plates seeded with *Escherichia coli* OP50 and incubated at 16 °C for 72 h. After incubation, the NGM plates containing larvae and eggs were washed with M9 buffer, and the supernatant was placed in 15-mL conical tubes. A bleaching solution (hypochlorite + NaOH) was further added, to kill the adult larvae. The eggs were placed in NGM plates and incubated again at 15 °C for 24 h. Later, the NGM plates containing the larvae at the L1/L2 stages were washed with M9 buffer, and the supernatant was transferred to NGM plates seeded with *E. coli* OP50 and incubated at 16 °C for 24 h. After synchronization, 20 µL of the NGM plate contents containing from 10 to 20 L4 stage larvae was added to each well of a 96-well flat-bottomed microplate and incubated at 16 °C for 72 h. The BRP crude hydroalcoholic extract was evaluated from 750 to 6000 μg/mL, and the isolated compounds oblongifolin B and guttiferone E were evaluated from 5.85 to 1500 μg/mL. DMSO was used as solvent (final concentration ≤ 1%).

Larvae were counted every 24 h for three consecutive days under an inverted microscope. Larvae with movement were considered alive and static even after touching they were considered dead. For each sample, the lowest concentration that was able to kill 50% of the larvae, called Lethal Concentration (LC_50_), was determined according to time.

### Statistical analysis

Individual experiments were performed in triplicate, and all the assays were performed a minimum of three times to confirm the reproducibility of the results. Differences between the means of the readings were compared by analysis of variance (one-way or two-way ANOVA) or Student’s t-test conducted with the software Graph Pad Prism 8.0 (Graph Pad Software). The *p* values ≤ than 0.05 were considered statistically significant.

## Supplementary Information

Supplementary Information.

## Data Availability

All data generated or analysed during this study are included in this published article (and its Supplementary Information files).
