# Supplementary material for: Potential in vitro anti-periodontopathogenic, anti-Chikungunya activities and in vivo toxicity of Brazilian red propolis
Source: Sci Rep. 2022 Dec 7;12:21165. doi: 10.1038/s41598-022-24776-4 (PMC9729292; doi:10.1038/s41598-022-24776-4)
Supplement: Supplementary file 1 — Supplementary Information. [file 41598_2022_24776_MOESM1_ESM.docx]

**SUPPLEMENTARY MATERIAL**

Potential in vitro anti-periodontopathogenic, anti-Chikungunya activities and in vivo toxicity of Brazilian Red Propolis

Nagela Bernadelli Sousa Silva^1,^ Jonathan Henrique de Souza^1^, Mariana Brentini Santiago^1^ Jhennyfer Rodrigues da Silva Aguiar^1^, Daniel Oliveira Silva Martins^1,2^, Rafael Alves da Silva^3^, Igor de Andrade Santos^1^, Jennyfer A. Aldana-Mejía^4^, Ana Carolina Gomes Jardim^1,2^, Reginaldo dos Santos Pedroso^5^, Sergio Ricardo Ambrósio^6^, Rodrigo Cássio Sola Veneziani^6^, Jairo Kenupp Bastos^4^, Regina Helena Pires^7^, Carlos Henrique Gomes Martins^1,*^

^1^ Federal University of Uberlândia, Institute of Biomedical Sciences (ICBIM) Uberlândia, Brazil

^2^ Sao Paulo State University, Institute of Biosciences, Letters and Exact Sciences (IBILCE), São José do Rio Preto, Brazil.

^3^ Federal University of Uberlândia, Faculty of Medicine (FAMED), Uberlândia, Brazil.

^4^ University of São Paulo (USP), Faculty of Pharmaceutical Sciences of Ribeirão Preto, Ribeirão Preto, Brazil.

^5^ Federal University of Uberlândia, Technical School of Health (ESTES) Uberlândia, Brazil.

^6^ University of Franca (UNIFRAN), Exact and Technological Sciences Nucleus, Franca, Brazil.

7 University of Franca (UNIFRAN), Postgraduate Program in Health Promotion, Franca, Brazil.

*[Corresponding Author:](mailto:corresponding.author@email.example)  carlos.martins2@ufu.br

**Supplementary Figure S1:** Chromatographic profile (275 nm) of Brazilian red propolis extract (A); hexane fraction (B); ethyl acetate fraction (C); dichloromethane fraction (D); *n*-butanol fraction (E). Numbers correspond to: 1, vestitol; 2, neovestitol; 3, medicarpin; 4, 7-*O*-methylvestitol; 5, guttiferone E/xanthochymol; and 6, oblongifolin B.


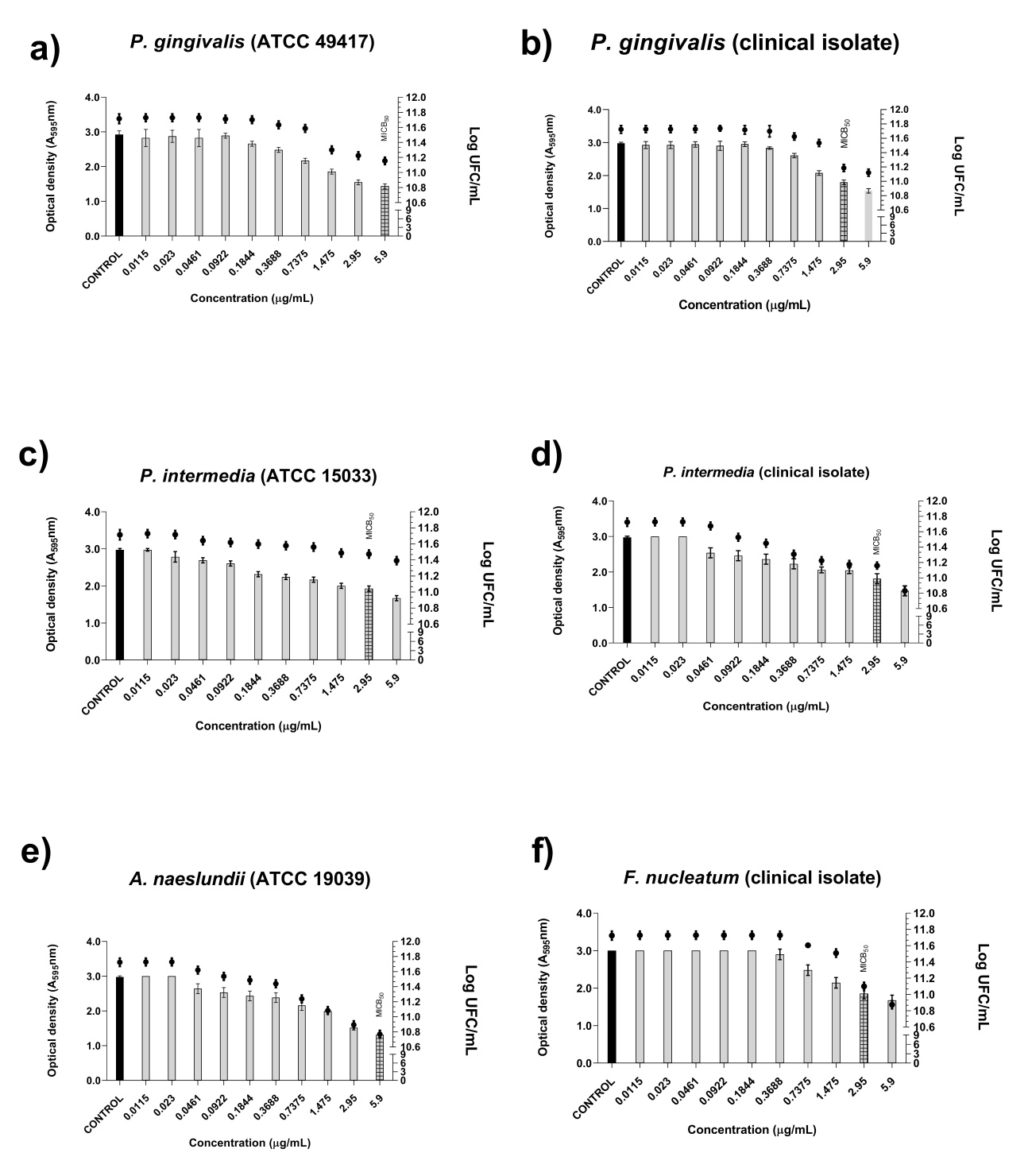


**Supplementary Figure S2**. Antibiofilm activity of metronidazole and number of viable cells in monospecies biofilms formed by ATCC strains and clinical isolates included in the study. **2A**: *P. gingivalis* (ATCC 49417). **2B**: *P. gingivalis* (clinical isolate). **2C**: *P. intermedia* (ATCC 15033). **2D**: *P. intermedia* (clinical isolate). **2E**: *A. naeslundii* (ATCC 19039). **2F**: *F. nucleatum* (clinical isolate).


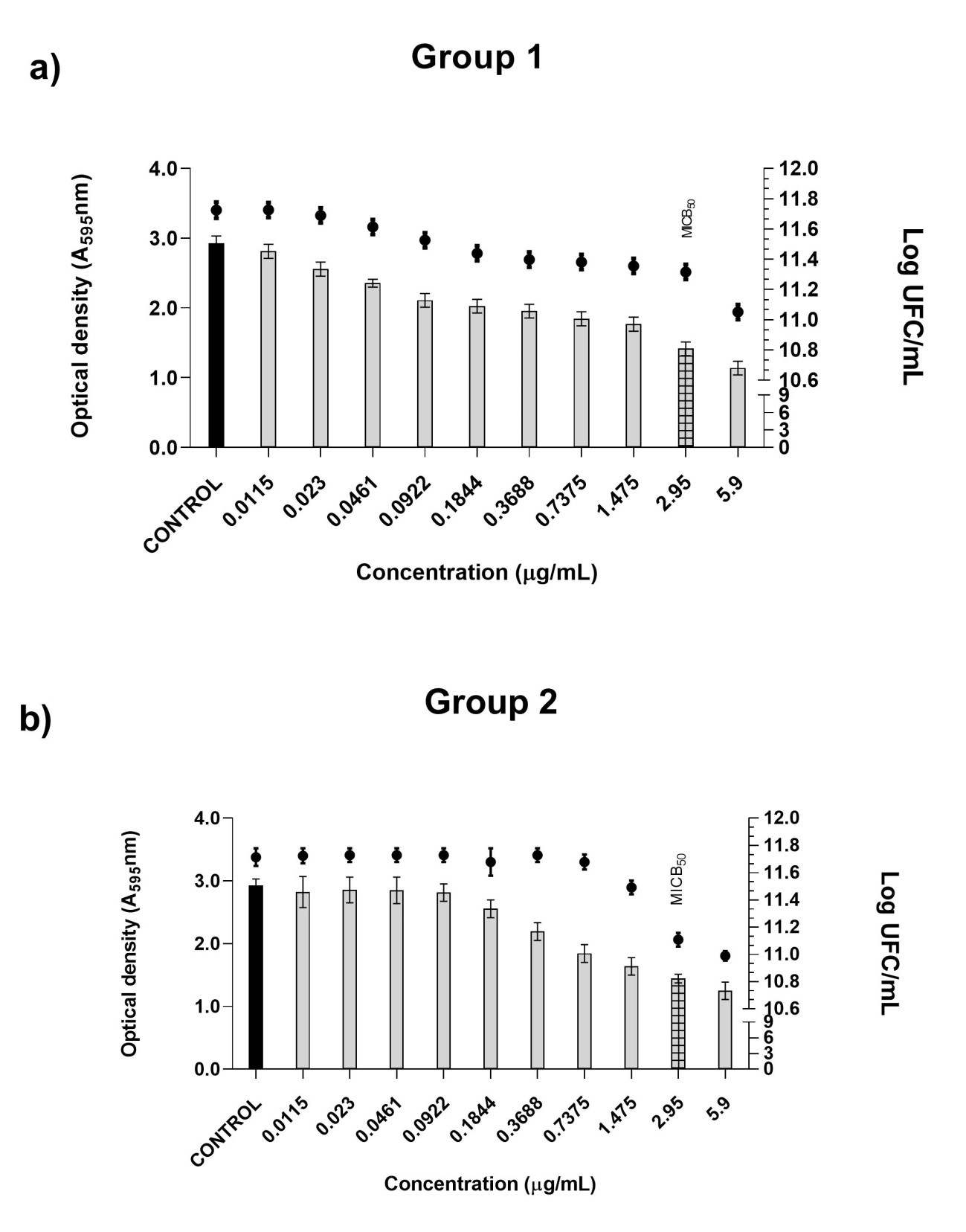


**Supplementary Figure S3**: Antibiofilm activity of samples of metronidazole and number of viable cells in multispecies biofilms. **3A**: Biofilm formed by standard strains. **3B**: Biofilm formed by clinical isolates.


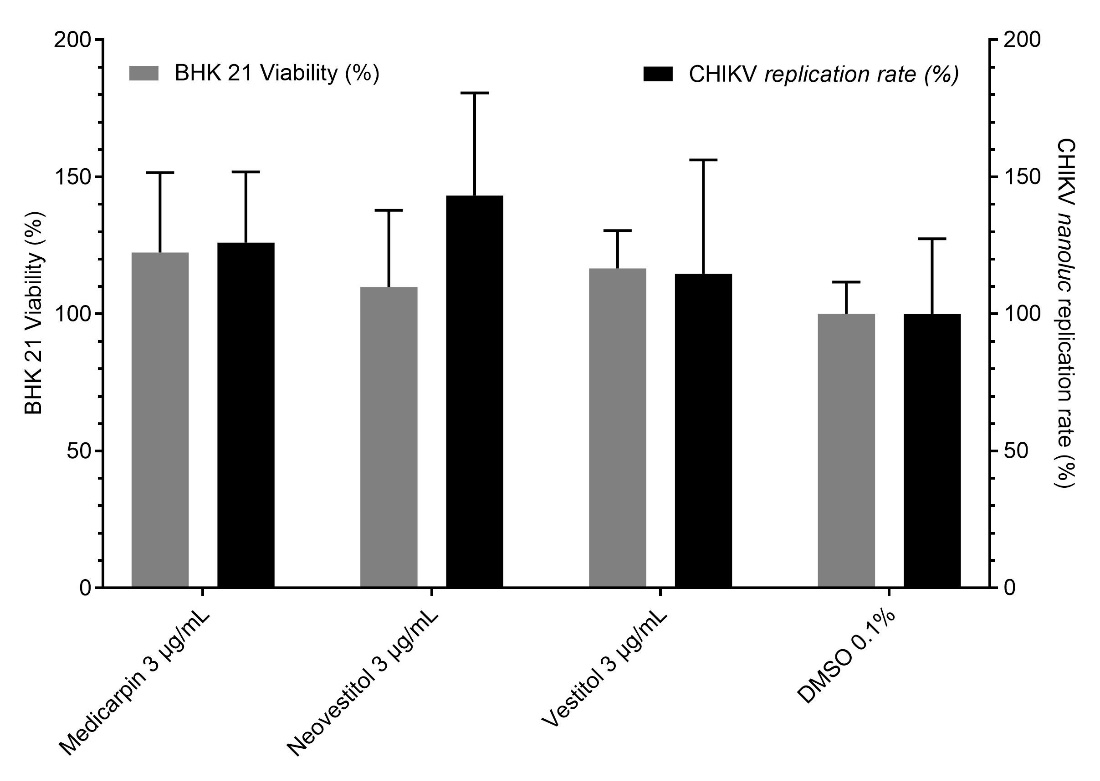


**Supplementary Figure S4. Effect of Medicarpin, Neovestitol amd Vestitol at 3 μg/mL on CHIKV replication *in vitro*.**
